# Estimating the risk of developing secondary hematologic malignancies in patients with T1/T2 prostate cancer undergoing diverse treatment modalities: A large population‐based study

**DOI:** 10.1002/cam4.4087

**Published:** 2021-06-29

**Authors:** Xiaofei Mo, Mingge Zhou, Hui Yan, Xueqin Chen, Yuetao Wang

**Affiliations:** ^1^ Department of Nuclear Medicine The Third Affiliated Hospital of Soochow University Changzhou Jiangsu China; ^2^ Changzhou Key Laboratory of Molecular Imaging Jiangsu China

**Keywords:** hematologic malignancy, prostate cancer, prostatectomy, radiation therapy

## Abstract

**Background:**

Patients with prostate cancer (PC) are at a high risk of developing secondary hematologic malignancies (SHMs) after radiation therapy (RT), while no study has assessed the relationship of different treatment modalities with the occurrence of SHMs after PC at early stage. This study aimed to investigate the risks of developing SHMs in patients with T1/T2 PC undergoing different treatment modalities.

**Methods:**

Patients with T1/T2 PC were identified from the Surveillance, Epidemiology, and End Results database. Competing risk regression (CRR) model was performed to evaluate the hazard ratios (HRs) of developing SHMs. As SHMs scarcely occur, the relative risk (RR) analysis was employed to compare the risks of different treatment modalities associating with the development of SHMs.

**Results:**

The CRR analysis showed that undergoing RT was associated with a higher risk of developing SHMs (external beam radiation therapy [EBRT]: HR = 1.21, 95% confidence interval [CI]: 1.10–1.34; radioactive implant [RI]: HR = 1.20, 95% CI: 1.06–1.36). As for different types of SHMs, EBRT, and RI were correlated with decreased risks of developing CLL (RR = 0.67, 0.72; 95% CI: 0.53–0.85, 0.54–0.96, respectively), but with the increased risks of developing NHL (RR = 1.18, 1.23; 95% CI: 1.02–1.35, 1.05–1.44, respectively); EBRT also showed increased risks of developing acute/ chronic myeloid leukemia (AML/CML, RR = 1.54, 1.56; 95% CI: 1.16–2.03,1.05–2.33, respectively); No increased risk of developing SHMs was detected in patients who only underwent prostatectomy.

**Conclusions:**

Although RT was found to be associated with the increased risks of developing SHMs in patients with T1/T2 PC, this finding cannot be extended to diverse types of SHMs. RT was correlated with the increased risks of the development of NHL, AML, and CML, but with the decreased risk of developing CLL. Prostatectomy did not increase the risk of developing SHMs.

## Graphical abstract

Our study used a large population‐based analysis to estimate the risk of different radiation therapy modalities for second hematologic malignancies developing after T1/2 prostate cancer. External beam radiation therapy and radioactive implant were correlated with increased risks of acute/chronic myeloid leukemia but decreased risk of chronic lymphocytic leukemia. The impact of radiation therapy on second hematologic malignancies developing after T1/2 prostate cancer was different in various types of hematologic malignancies.

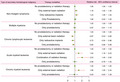

## 1 INTRODUCTION

Prostate cancer (PC) is the most common malignancy in middle‐aged and elderly men, accounting for 10% of cancer‐related deaths in the United States according to the statistics published by the American Cancer Society.^1^, ^2^, ^3^ For patients with T1/T2 PC, radiation therapy (RT) and prostatectomy are the main therapeutic options, including external beam radiation therapy (EBRT), radioactive implant (RI), and combination of RT with prostatectomy.^4^ The PC‐specific survival of patients who underwent RT has markedly attracted scholars’ attention.^5^, ^6^ However, it was frequently reported that RT is associated with a high risk of secondary hematologic malignancies (SHMs) in patients with diverse types of cancer.^7^, ^8^, ^9^, ^10^, ^11^, ^12^ This can be related to the fact that RT enhances the fitness of clonal hematopoietic stem cells, which can influence outcome through progression to hematologic malignancies and through cell nonautonomous effects on solid tumors.^13^, ^14^

Although a number of scholars have concentrated on the association between RT and development of SHMs in patients with early‐stage PC, few researches have investigated the risk of developing SHMs in such patients undergoing various therapies. The present study aimed to assess the risk of developing SHMs in patients with T1/2 PC who underwent different treatment modalities.

## 2 METHODS

### 2.1 Data collection

The Surveillance, Epidemiology, and End Results (SEER) database covers approximately 30% of the US population and provides complete cancer patient data, including demographic, clinical information, and follow‐up data. This database is updated annually by the National Center for Health Statistics.^15^ It also provides incidence, survival, and mortality data for histopathologic cancer subtypes. We chose 18 registry research datasets (2000–2015, with additional treatment fields; November 2017) in the SEER database to identify cases with T1/T2 PC.

### 2.2 Inclusion and exclusion criteria

The inclusion criteria were as follows: (a) PC was the first primary cancer, performing diagnosis according to the International Classification of Diseases for Oncology, third edition (ICD‐O‐3; particularly C61.9) and with T1‐T2M0 stage according to the American Joint Committee on Cancer (AJCC) stage system; (b) conducting diagnosis between 2004 and 2010.

The SHMs as one of the outcomes after PC diagnosis were identified according to the ICD‐O‐3 morphology codes 959–994, which included acute lymphocytic leukemia, acute monocytic leukemia, acute myeloid leukemia (AML), chronic lymphocytic leukemia (CLL), chronic myeloid leukemia (CML), Hodgkin lymphoma, multiple myeloma (MM), non‐Hodgkin lymphoma (NHL), and other leukemias.

### 2.3 Statistical analysis

After data entry, data were manually and statistically checked as a part of the data cleaning process. Then, descriptive statistics were calculated for patients’ demographic and clinical data at baseline. The Chi‐square test was utilized to analyze categorical variables; the skewness and kurtosis were used to evaluate the distribution of continuous data, and the Kruskal–Wallis test was employed to analyze those data. A two‐sided *p*‐value <0.05 was considered statistically significant.

### 2.4 Competing risk regression model

We used a competing risk regression (CRR) model to evaluate the hazard ratios (HRs) of SHMs. Patients’ demographic and clinical data were imported into the model, which included patients’ age at the time of diagnosis of PC, marital status, race, T and N stages of the AJCC staging system, prostate‐specific antigen (PSA) level, receiving chemotherapy or not, Gleason's score and treatment modality. Among them, age at the time of diagnosis was considered as a continuous variable, and the PSA level and the Gleason's score were categorized by their clinical implications (i.e., PSA level at the range of 0–4 is normal; a Gleason's score of <7 represents a low‐grade cancer, a Gleason's score equal to 7 indicates a medium‐grade cancer, and a Gleason's score of >7 represents a high‐grade cancer). The variable of treatment modality included EBRT, RI, combination of EBRT and RI, prostatectomy, and combination of RT and prostatectomy, whereas no RT or prostatectomy was taken as reference into account. Patients who were alive at the last follow‐up were regarded as censored patients, and the development of other malignancies and death before the occurrence of SHMs were considered as competing risks. Cases from autopsy/death certification reports and not in active follow‐up were lack of survival periods, we assigned their survival time as the mean values of the corresponding outcomes in our study.

All the variables were firstly imported into the univariate CRR analysis, and then, the variables with significant differences were imported into the multivariate CRR analysis. After that, multicollinearity was assessed using variance inflation factor (VIF), measuring the inflation in the variances of the parameter estimates due to multicollinearity potentially caused by the correlated predictors.

### 2.5 Relative risk regression analysis of the risks of different treatment modalities associating with the development of SHMs

As SHMs scarcely occur, the relative risk (RR) regression analysis was employed to compare the risks of different treatment modalities associating with the development of SHMs. In the present study, therapeutic methods were taken as exposure, SHMs as outcomes, and RR was calculated as follows,^16^, ^17^

$$\text{RR}=\frac{O_{1}/N_{1}}{O_{2}/N_{2}}\times 100\%,$$

where *O* represents the number of observations in each cohort and *N* denotes the person‐year at risk in each cohort

As the incidence rate was significantly different in each cohort, *O* was adjusted to eliminate the difference with the reference cohort,^16^ which was formulated as follows,

$$\frac{O_{1}}{O_{a}}=\frac{E_{1}/N_{1}}{E_{R}/N_{R}}\times 100\%,$$

where *E* represents the number of expectations in each cohort.

The subscripts “a” and “R” denote “adjusted” and “reference cohort,” respectively.

We utilized multiple primary‐standardized incidence ratios (MP‐SIRs) in the SEER database to calculate the RR value. The values of *E* parameter could be achieved according to patients’ age, race, gender, and calendar time‐specific incidence rate by stratum‐specific person‐years of follow‐up. The 95% confidence intervals (CIs) and SIRs were derived from SEER*stat with the quantile approximation of the Chi–square distribution.^18^, ^19^ A RR value with 95% CI >1 or <1 was considered statistically significant. Data, in the present study, were analyzed by the R 3.6.3 programming language. The cmprsk package in the R 3.6.3 programming language was used to establish the competing risk model.

## 3 RESULTS

### 3.1 Patients’ demographic and clinical data

A total of 288,400 patients with T1/T2 PC met the defined criteria, of whom 3479 patients experienced development of SHMs (Table 1). Among them, 1597 (45.9%) patients experienced development of SHMs into NHL, 723 (20.8%) into MM, 485 (13.9%) into CLL, 335 (9.6%) into AML, 164 (4.7%) into CML, and 175 (5.0%) into other SHMs. Therefore, NHL, MM, and CLL were the top three frequently developed SHMs in patients with T1/2 PC.

**TABLE 1:** Demographic, clinical data and outcomes of patients with T1/2 PC

Variable	Subgroup	Overall cohort (n = 288,400)	No RT or prostatectomy (n = 66,132, 22.93%)	Only EBRT (n = 64,020, 22.20%)	Only RI (n = 29,115, 10.23%)	Combination of EBRT/RI/radioisotopes (n = 15,695, 5.44%)	Only prostatectomy (n = 108,305, 37.55%)	Prostatectomy with RT (n = 4737, 1.64%)	*p*‐value
Age at diagnosis		66.0 (9.32)	70.0 (10.07)	68.8 (7.85)	65.7 (7.83)	65.9 (7.97)	62.0 (8.55)	65.6 (8.79)	<0.001
Race	White	226888 (78.67)	49293 (74.54)	48552 (75.84)	24343 (82.49)	11683 (74.44)	89347 (82.50)	3670 (77.48)	<0.001
	Black	42283 (14.66)	10570 (15.98)	10794 (16.86)	3717 (12.60)	3217 (20.50)	13201 (12.19)	784 (16.55)	
	Asian or Pacific Islander	13225 (4.59)	3034 (4.59)	3568 (5.57)	1161 (3.93)	689 (4.39)	4518 (4.17)	255 (5.38)	
	American Indian/Alaska Native	857 (0.30)	252 (0.38)	200 (0.31)	73 (0.25)	33 (0.21)	285 (0.26)	14 (0.30)	
	Unknown	5147 (1.78)	2983 (4.51)	906 (1.420)	217 (0.74)	73 (0.47)	954 (0.88)	14 (0.30)	
Marital status	Single/divorce/widow	59646 (20.68)	14598 (22.07)	14957 (23.36)	5945 (20.18)	3144 (20.03)	19953 (18.42)	1040 (21.95)	<0.001
	Married	196066 (67.98)	33159 (50.14)	43481 (67.92)	21723 (73.61)	11593 (73.86)	82635 (76.30)	3475 (73.36)	
	Unknown	32688 (11.33)	18375 (27.76)	5582 (8.72)	1834 (6.21)	958 (6.10)	5717 (5.28)	222 (4.69)	
T (AJCC 6th) status	Ⅰ	120013 (41.61)	36371 (55.00)	40224 (62.83)	20498 (69.46)	9902 (63.09)	11054 (10.21)	1964 (41.46)	<0.001
	Ⅱ	168387 (58.39)	29761 (45.00)	23796 (37.17)	9013 (30.54)	5793 (36.91)	97251 (89.79)	2773 (58.54)	
N (AJCC 6th) status	0	283882 (98.43)	64640 (97.74)	62931 (98.30)	29192 (98.92)	15541 (99.02)	107008 (98.80)	4570 (96.47)	<0.001
	Ⅰ	1557 (0.54)	466 (0.70)	391 (0.61)	17 (0.06)	44 (0.28)	528 (0.49)	111 (2.34)	
	X	2961 (1.03)	1026 (1.55)	698 (1.09)	302 (1.02)	110 (0.70)	769 (0.71)	56 (1.18)	
Chemotherapy	No/Unknown	287635 (99.73)	65924 (99.69)	63765 (99.60)	29467 (99.85)	15638 (99.64)	108145 (99.85)	4696 (99.13)	<0.001
	Yes	765 (0.27)	208 (0.31)	255 (0.40)	44 (0.15)	57 (0.36)	160 (0.15)	41 (0.87)	
PSA, ng/mL (IQR)		6.1 (4.6–9.2)	7.1 (4.9–12.1)	7.1 (5.1–11.1)	5.7 (4.5–7.6)	6.3 (4.7–9.9)	5.4 (4.2–7.5)	6.6 (4.6–11)	<0.001
	0–4	38718 (13.43)	6372 (9.64)	5918 (9.24)	4348 (14.73)	1906 (12.14)	19448 (17.96)	726 (15.33)	<0.001
	>4	214345 (74.32)	45711 (69.12)	55452 (86.62)	23473 (79.54)	12960 (82.57)	73291 (67.67)	3458 (73.00)	
	Unknown	35337 (12.25)	14069 (21.24)	2650 (4.14)	1690 (5.73)	829 (5.28)	15566 (14.37)	553 (11.67)	
Gleason's score (IQR)		6 (6–7)	6 (6–7)	7 (6–7)	6 (6–7)	7 (6–7)	7 (6–7)	7 (6–7)	<0.001
	0–4	2440 (0.85)	1020 (1.54)	276 (0.43)	224 (0.76)	49 (0.31)	831 (0.77)	40 (0.84)	<0.001
	5–7	203118 (70.43)	41441 (62.66)	41727 (65.18)	24141 (81.80)	11016 (70.19)	81733 (75.47)	3060 (64.60)	
	7–10	25123 (8.71)	6779 (10.25)	8649 (13.51)	899 (3.05)	2211 (14.09)	5820 (5.37)	765 (16.15)	
	Unknown	57719 (20.01)	16892 (25.54)	13368 (20.88)	4255 (14.7)	2419 (15.41)	19921 (18.39)	872 (18.41)	
Outcomes	Alive	211723 (73.41)	40389 (61.07)	43830 (68.46)	22474 (76.15)	11705 (74.58)	90002 (83.10)	3323 (70.15)	<0.001
	Other second cancer	29031 (10.07)	6874 (10.39)	7806 (12.19)	3384 (11.47)	1797 (11.45)	8635 (7.97)	535 (11.29)	
	Death	44342 (15.38)	18100 (27.37)	11414 (18.1)	3267 (11.07)	1979 (12.61)	8663 (8.00)	813 (17.16)	
	NHL	1597 (0.55)	343 (0.52)	411 (0.64)	201 (0.68)	95 (0.61)	516 (0.48)	31 (0.65)	
	MM	723 (0.25)	178 (0.27)	187 (0.29)	76 (0.26)	49 (0.31)	219 (0.20)	14 (0.30)	
	CLL	485 (0.17)	153 (0.23)	101 (0.16)	52 (0.18)	26 (0.17)	150 (0.14)	3 (0.06)	
	AML	335 (0.12)	67 (0.10)	110 (0.17)	36 (0.12)	34 (0.22)	76 (0.07)	12 (0.25)	
	CML	164 (0.06)	28 (0.04)	55 (0.09)	21 (0.07)	10 (0.06)	44 (0.04)	6 (0.13)	

Note

The PSA value and Gleason's score is presented as median (IQR), age at diagnosis is mean (SD),while other variables are frequency (%).

Abbreviations: AJCC, American Joint Committee on Cancer; AML, acute myeloid leukemia; CLL, chronic lymphocytic leukemia; CML, chronic myeloid leukemia; EBRT, external beam radiation; MM, multiple myeloma; NHL, non‐Hodgkin lymphoma; PSA, prostate‐specific antigen; RI, radioactive implants; RT, radiation therapy; SHM, second hematological malignancy.

According to the treatment modality, patients were divided into 6 groups, including no RT or prostatectomy (n = 66132, 22.93%), only EBRT (n = 64020, 22.20%), only RI (n = 29115, 10.23%), combination of EBRT/RI/radioisotopes (n = 15695, 5.44%), only prostatectomy (n = 108305, 37.55%), and combination of RT and prostatectomy (n = 4737, 1.64%). The patients’ characteristics in each group are summarized in Table 1.

### 3.2 CRR analysis of the development of SHMs

As shown in Table 2, age at the time of diagnosis of PC, marital status, and RT were associated with the risk of the development of SHMs by multivariate CRR analysis. More specifically, elderly patients were at a higher risk of the development of SHMs than non‐elderly patients (HR = 1.03, 95% CI: 1.02–1.04); married patients were at a higher risk of the development of SHMs compared with unmarried patients (HR = 1.30, 95% CI: 1.19–1.42). Besides, patients who received EBRT, RI, combination of EBRT and RI, and combination of prostatectomy and RT were at a higher risk of the development of SHMs than those who did not receive RT or prostatectomy (HR = 1.21, 95% CI: 1.10–1.34; HR = 1.20, 95% CI: 1.06–1.36; HR = 1.27, 95% CI: 1.10–1.48 and HR = 1.36, 95% CI: 1.07–1.74, respectively). However, patients who underwent prostatectomy were not at a higher risk of development of SHMs than those who did not undergo RT or prostatectomy. The VIFs of the variables are presented in Table 2, and a VIF <2 indicated that multicollinearity was not existed among these factors. The results of univariate CRR analysis are presented in Data S1.

**TABLE 2:** The results of multivariable competing risk regression analysis of developing SHMs in patients with T1/2 PC

Variables	HR	95% CI lower‐upper	*p*‐value	VIF
Age at diagnosis	1.03	1.02–1.04	<0.001	0.867
Marital status				
Single/divorce/widow	1.00			
Married	1.30	1.19–1.42	<0.001	0.722
Unknown	1.16	1.02–1.32		0.697
Race				/
White	1.00			
Black	0.98	0.89–1.09	0.752	
Asian or Pacific Islander	0.87	0.74–1.03	0.105	
American Indian/Alaska Native	0.70	0.33–1.56	0.337	
Unknown	0.05	0.01–0.16	<0.001	
T (AJCC 6th) status				/
Ⅰ	1.00			
Ⅱ	1.02	0.95–1.10	0.615	
Therapy modality				
No RT or prostatectomy	1.00			
Only EBRT	1.21	1.10–1.34	<0.001	0.628
Only RI	1.20	1.06–1.36	0.003	0.733
Combination of EBRT/RI/radioisotopes	1.27	1.10–1.48	0.002	0.829
Only prostatectomy	1.02	0.92–1.12	0.755	0.505
RT with prostectomy	1.36	1.07–1.74	0.012	0.937

Abbreviations: AJCC, American Joint Committee on Cancer; CRR, competing risk regression analysis; EBRT, external beam radiation; HR, hazard ratio; RI, radioactive implants; VIF, variance inflation factor.

### 3.3 RRs of the development of SHMs in patients with T1/T2 PC undergoing diverse treatment modalities

Table 3 presents the RRs of the development of SHMs in patients with T1/T2 PC undergoing diverse treatment modalities. It was found that undergoing EBRT was associated with a higher risk of NHL, AML, and CML with RRs of 1.18 (95% CI: 1.02–1.35), 1.54 (95% CI: 1.16–2.03), and 1.56 (95% CI: 1.05–2.33), respectively, while that was correlated with an attenuated risk of CLL (RR = 0.67, 95% CI: 0.53–0.85). In addition, undergoing RI was associated with a higher risk of NHL with RR of 1.23 (95% CI: 1.05–1.44) and with a lower risk of CLL (RR = 0.72, 95% CI: 0.54–0.96), and undergoing combination of EBRT/RI/radioisotopes was correlated with a higher risk of AML (RR = 2.03, 95% CI: 1.40–2.93). Moreover, patients who underwent prostatectomy was associated with an attenuated risk of CLL (RR = 0.80, 95% CI: 0.66–0.98) and did not show correlation with developing other types of SHMs. The SIRs of each cohort are presented in Data S2.

**TABLE 3:** RRs of various types of SHMs after undergoing different treatment modalities

SHMs	Therapy modality	O	E	Person‐year at risk	Oa	RR	95% CI lower‐upper
NHL	No prostatectomy or RT	368	427.81	412709.81	Reference		
	Only EBRT	463	458.05	441002.69	462.0782	**1.18**	**1.02–1.35**
	Only RI	226	213.59	226584.32	248.5212	**1.23**	**1.05–1.44**
	Combination of EBRT/RI/radioisotopes	99	102.85	111976.09	111.7281	1.12	0.91–1.38
	Only Prostatectomy	550	605.05	799191.88	753.0583	1.06	0.93–1.20
	RT with prostatectomy	11	12.83	38705.21	34.39867	1.10	0.70–1.41
MM	No prostatectomy or RT	187	181.01	412709.81	Reference		
	Only EBRT	207	198.48	441002.69	201.72	1.01	0.83–1.23
	Only RI	82	87.77	226584.32	92.84	0.90	0.71–1.16
	Combination of EBRT/RI/radioisotopes	50	46.41	111976.09	52.91	1.04	0.77–1.41
	Only prostatectomy	245	245.64	799191.88	349.60	0.97	0.81–1.15
	RT with prostatectomy	6	8.14	38705.21	12.51	0.71	0.40–1.26
CLL	No prostatectomy or RT	159	144.82	412709.81	Reference		
	Only EBRT	112	152.44	441002.69	113.70	**0.67**	**0.53–0.85**
	Only RI	56	70.8	226584.32	62.89	**0.72**	**0.54–0.96**
	Combination of EBRT/RI/radioisotopes	29	34.13	111976.09	33.39	0.77	0.53–1.12
	Only prostatectomy	174	197	799191.88	247.70	**0.80**	**0.66–0.98**
	RT with prostatectomy	3	3.43	38705.21	11.87901		0.44–1.44
AML	No prostatectomy or RT	80	95.08	412709.81	Reference		
	Only EBRT	132	102.15	441002.69	131.29	**1.54**	**1.16–2.03**
	Only RI	49	45.5	226584.32	56.22	1.28	0.91–1.80
	Combination of EBRT/RI/radioisotopes	38	22.3	111976.09	43.96	**2.03**	**1.40–2.93**
	Only prostatectomy	102	122.05	799191.88	153.87	0.99	0.86–1.30
	RT with prostatectomy	9	6.15	38705.21	13.04912	1.74	0.97–3.12
CML	No prostatectomy or RT	38	38.84	412709.81	Reference		
	Only EBRT	63	41.16	441002.69	63.52	**1.56**	**1.05–2.33**
	Only RI	27	18.65	226584.32	30.87	1.48	0.92–2.38
	Combination of EBRT/RI/radioisotopes	11	9.1	111976.09	12.74	1.24	0.65–2.33
	Only prostatectomy	47	51.47	799191.88	68.68	0.93	0.63–1.38
	RT with prostatectomy	0	0.64	38705.21	0	/	/

Bold fonts: *p *< 0.05.

Abbreviations: AML, acute myeloid leukemia; CI, confidence intervals; CLL, chronic lymphocytic leukemia; CML, chronic myeloid leukemia; E, expectations; EBRT, external beam radiation; MM, multiple myeloma; NHL, non‐Hodgkin lymphoma; O, observations; Oa, observations(adjusted); RI, radioactive implants; RR, relative risk; RT, radiation therapy; SHM, second hematological malignancy.

## 4 DISCUSSION

RT remains an important component of cancer treatment with approximately 50% of all cancer patients receiving RT during their course of disease.^20^ A number of scholars pointed out that RT is effective for improving cancer‐specific survival rate in various malignancies, especially in T1/2 PC.^21^, ^22^, ^23^ However, it has been frequently reported that the risk of the development of SHMs after RT is noteworthy and whether RT can attenuate the risk of the development of SHMs remains controversial.^24^, ^25^, ^26^ In the present study, a large population‐based database was used to analyze the association between RT and the development of SHMs in patients with T1/2 PC.

Regarding the development of SHMs in patients with T1/2 PC, it was noted that NHL, MM, and CLL were the top three frequently developed SHMs. While some studies have showed thyroid cancer was noticeably enhanced the development of AML and CML after radioiodine treatment,^7^ and breast cancer could significantly intensify the development of AML and NHL after RT.^10^ According to the report of the American Cancer Society published in 2020, the most frequently occurred SHMs in the United States were NHL, MM, and CLL,^3^ which was consistent with the result of the current research, but not totally coincide with thyroid cancer and breast cancer as mentioned above. Therefore, it is necessary to focus on the incidence of NHL, MM, and CLL in T1/T2 PC patients. As mentioned earlier, RT was found to be associated with a high incidence of secondary AML and CML in some malignancies, a number of scholars demonstrated that genotoxic stress, ribosome biogenesis stress, and inflammation from RT might increase the risk of transformation from clonal hematopoiesis to a myeloid malignancy included AML and CML,^27^, ^28^ which highlighted the necessity of estimation of the risks of development of AML and CML in T1/T2 PC patients.

Although a number of previous studies have shown that RT was associated with a higher risk of the development of SHMs, the models and factors included in those studies were not comprehensive,^8^, ^11^, ^12^ and we, in the present research, used CRR analysis to assess the risk of the development of SHMs, which included factors related to PC patients’ demographic and clinical characteristics. We found that the risk of developing SHMs was elevated with the increase of age, married patients were at a higher risk compared with unmarried ones, and undergoing RT was also associated with the increased risk of developing SHMs. It has reported that age‐related clonal hematopoiesis was a common condition that was associated with increases in the risk of hematologic cancer, where a single mutant hematopoietic stem or progenitor cell contributes to a significant, measurable clonal proportion of mature blood lineages.^29^ Evolution of mutant clonal hematopoiesis with age predisposes the elderly to myelodysplastic syndromes (MDS), AML, and other aging‐associated diseases.^27^, ^28^ As for marital status of patients with PC, some scholars have reported that married patients had better prognosis than unmarried, but for the development of SHMs,^30^, ^31^ this result was opposite in our study, and it is expected that further researches could explain these results.

Several previously conducted studies have demonstrated that RT was associated with a high incidence of AML and CML,^7^, ^8^, ^10^, ^25^ and we therefore attempted to estimate the risks of developing these SHMs in patients with T1/2 PC. The RR is the ratio of risk of an event in one group (e.g., exposed group) to the risk of the event in the other group (e.g., nonexposed group). Adjustment for baseline covariates in the analysis of randomized controlled trials can lead to a substantial increase in power when the covariates are highly prognostic.^16^, ^17^, ^32^ In the present study, values of *E* parameter could be achieved according to the patients’ age, race, and calendar time‐specific incidence rate by stratum‐specific person‐years of follow‐up.

Regarding the RRs in the current study, RT was found to be associated with a significantly increased risk of developing AML and CML and a slightly increased risk of developing NHL than those who did not receive RT or prostatectomy, whereas patients who only underwent prostatectomy did not show the correlations of increased risk of developing SHMs. Although a large population‐based database was utilized in the current study, it was revealed that the influence of RT on the development of SHMs was different, the incidence of AML and CML was more sensitive to RT, and the decreased risk of developing CLL might be attributed to the treatment for PC.

Some limitations existed in the present study should be presented. First, the RT protocols for PC patients were not existed in the SEER database, thus, we could not take the radiation dosage into account. Second, some patients’ demographic and clinical data were unidentifiable and the present retrospective study was therefore vulnerable to ascertainment bias.^7^ Third, the selection bias was found due to the retrospective nature of this study. Despite the above‐mentioned limitations, utilization of a large population‐based database enhanced the reliability of the results. However, further research is warranted to eliminate these limitations and to confirm our findings.

## 5 CONCLUSIONS

In summary, NHL, MM, and CLL were the top three frequently developed SHMs in patients with T1/2 PC. Besides, undergoing RT was associated with the increased incidence of NHL, AML, and CML, while with the decreased incidence of CLL, and no significant association was detected between undergoing RT and the incidence of MM. Moreover, prostatectomy did not significantly correlate with the increased risk of developing SHMs.

## 6 ETHICS APPROVAL AND CONSENT TO PARTICIPATE

We received permission from the National Cancer Institute, US to access the research data file in the SEER program (reference number 13610‐Nov2019). Ethics approval was not applicable because SEER data is publicly available and without specific identifiers.

## DATA AND MATERIAL AVAILABILITY STATEMENT

The datasets analyzed during the current study are available in the SEER repository (https://seer.cancer.gov/). The databases are public access.

## CONFLICT OF INTEREST

The authors have no conflict of interest.

## AUTHOR CONTRIBUTIONS

XM, MZ, and YW conceived the study design and analytical concept. XM conducted the data acquisition, performed the statistical analyses and drafted the manuscript. HY assisted with collection of data and interpretation. XC participated in data interpretation and revision of manuscript. XM and MZ contributed to the interpretation of the results and the critical revision of the manuscript. The authors participated in the revision of the manuscript and approved the final manuscript.

## Supporting information

Data S1

Data S2
